# CRBP-TS - evaluation of a home-based training and health care program for colorectal, breast, and prostate cancer using telemonitoring and self-management: study protocol for a randomized controlled trial

**DOI:** 10.1186/s13102-021-00244-w

**Published:** 2021-02-23

**Authors:** Roberto Falz, René Thieme, Uwe Tegtbur, Christian Bischoff, Christian Leps, Peter Hillemanns, Kay Kohlhaw, Jürgen Klempnauer, Florian Lordick, Jens-Uwe Stolzenburg, Bahriye Aktas, Jürgen Weitz, Ulrich Bork, Pauline Wimberger, Christian Thomas, Ronald Biemann, Boris Jansen-Winkeln, Antina Schulze, Ines Gockel, Martin Busse

**Affiliations:** 1 Institute of Sport Medicine and Prevention, University Leipzig, Marschnerstraße 29a, 04109 Leipzig, Germany; 2 Department of Visceral, Transplant, Thoracic and Vascular Surgery, University Hospital Leipzig, Leipzig, Germany; 3 Institute of Sport Medicine, Hannover Medical School, Hannover, Germany; 4 Department of Gynecology and Obstetrics, Hannover Medical School, Hannover, Germany; 5 Department of Surgery, Sana Hospitals “Leipziger Land”, Borna, Germany; 6 Department of General, Visceral and Transplant Surgery, Hannover Medical School, Hannover, Germany; 7 Department of Oncology, Gastroenterology, Hepatology, Pulmonology and Infectious Diseases, University Hospital Leipzig, Leipzig, Germany; 8 University Cancer Center Leipzig, University Hospital Leipzig, Leipzig, Germany; 9 Department of Urology, University Hospital Leipzig, Leipzig, Germany; 10 Department of Gynaecology, University Hospital Leipzig, Leipzig, Germany; 11 Department of Visceral-, Thoracic and Vascular Surgery, University Hospital Carl Gustav Carus, Technische Universität Dresden, Dresden, Germany; 12 National Center for Tumor Diseases (NCT), Partner site Dresden, Dresden, Germany; 13 Department of Gynecology and Obstetrics, University Hospital Carl Gustav Carus, Technische Universität Dresden, Dresden, Germany; 14 Department of Urology, University Hospital Carl Gustav Carus, Technische Universität Dresden, Dresden, Germany; 15 Institute of Laboratory Medicine, Clinical Chemistry and Molecular Diagnostics, University Hospital Leipzig, Leipzig, Germany

**Keywords:** Cancer, Home-based exercise, Oxygen uptake, Counseling

## Abstract

**Background:**

Physical training is recommended in various national and international guidelines for patients with cancer. Observational studies have shown that physical activity leads to reduced recurrence and mortality rates by 20–40% in colorectal, breast, and prostate cancer. Despite existing evidence, a systematic care structure is still lacking. The primary aim of this study is to implement and evaluate an online training platform to strengthen physical performance and patient empowerment after cancer surgery.

**Methods:**

The evaluation will be conducted as a prospective multicenter randomized controlled trial with three subgroups (colorectal-, breast-, and prostate cancer). Each group will include 100 patients (total 300 patients including dropouts; clinical stages T1–3 and/or N+; M0 after surgery intervention) and the primary endpoint (13% increase in the maximal oxygen consumption during exercise) will be examined. The intervention group will receive a 6-month home-based online training (2–3 times per week strength-endurance training using video presentations), bidirectional activity feedback information, online communication, and online counseling. The control group (usual care) will be advised lifestyle improvement. In-hospital testing will be performed before, during, and after the intervention. In addition to cardiopulmonary capacity, tumor specific diagnostics (liquid biopsy, depression and fatigue assessment, metabolic and endothelial screening) will be applied.

**Discussion:**

Due to the increasing incidence of cancer, associated with considerable mortality, morbidity and impaired quality of life, there is an imperative requirement for improved cancer care, of which structured physical training may become an integral component.

**Trial registration:**

DRKS-ID: DRKS00020499; Registered 17 March 2020.

## Background

Prostate cancer and breast cancer are the most common malignant tumors in men and women, respectively with approximately 60,000–70,000 new cases each per year in Germany. Colorectal carcinoma affects women and men with an increasing incidence of approximately 60,000 new cases per year [1]. The etiology of these malignancies is multifactorial. It includes genetic, environmental, and lifestyle factors such as nutrition, alcohol consumption, and smoking. Physical inactivity can increase cancer mortality [2]. Therefore, regular exercise is one of the most effective cancer prevention mechanisms [3–5]. Exercise also has a positive effect on the prevention of cancer recurrence. Regular training has been shown to result in a reduction of approximately 20–40% in the risk for colorectal, breast, and prostate cancer [6–12]. A strong positive correlation has been demonstrated between weekly energy consumption [10] and cardiorespiratory fitness [13] and tumor-related mortality rate [14]. In addition, regular training has been shown to improve cancer-related symptoms such as fatigue, muscle loss, metabolic syndrome and diabetes, and chronic cardiovascular inflammation. The benefits of regular training are also applicable to comorbidities such as anxiety disorders, depression [15], cognitive deficits, and dementia [16], which can further impair quality of life and therapeutic adherence of patients [17].

The World Health Organization, the European Society for Medical Oncology and the American Cancer Society recommend at least 150 min of moderate exercise or 75 min of vigorous exercise per week for all adults for primary and tertiary cancer prevention.

However, in patients undergoing chemotherapy, chemoradiotherapy, or cancer surgery, these recommendations are often not fully applicable and must be adapted to the patients’ individual level of performance. Consequently, training mode, training frequency, and intensity must be individually set according to personal performance and adapted according to the postsurgical conditions. Further individual adaptions are also necessary in the course of the therapy [18]. Essential factors to maintain the patients’ adherence to the training program include ongoing personal support, consideration of patients’ individual capability with thorough online training adjustment, and a bidirectional communication with the counseling team.

Training-associated decrease in cancer-related mortality rates is multifactorial. Several pathways and mechanisms have been discussed previously, which include regulation of tumor growth via phosphoinositide 3-kinase pathway [19], microRNA regulation [19, 20], decrease in circulating tumor cells [21], more consistent application of chemotherapy [22], attenuated epithelial-mesenchymal transition and tumor cell migration [23], and altered DNA methylation [24].

The available literature is primarily based on questionnaire surveys, instructions for lifestyle changes, and qualitative implementation of the training programs with subjective assessment of the intensity [25]. Despite the strong evidence and the preventive and regenerative efficiency of physical training, systematic implementation or adequate care structure are lacking in cancer therapy.

The **C**olo**R**ectal, **B**reast, and **P**rostate Cancer - **T**elemonitoring and **S**elf-management project (CRBP-TS) addresses these issues. The study aims to combine online-supported training, automated recording of activity and performance parameters, cross-sectoral bidirectional data evaluation, and online communication based on an electronic case file, which is accessible to patients as well as physicians. The objective of this study is to implement and evaluate an online training platform to strengthen physical performance and patient empowerment after cancer surgery. The primary outcome is 13% increase in maximal oxygen consumption during exercise (VO_2_ max) in the intervention group (IG) when compared with the control group (CG) after training for 6 months.

## Methods

### Study design and ethical approval

This prospective multicenter randomized controlled trial is organized by the Institute of Sport Medicine and Prevention, University of Leipzig, Germany and the Department of Visceral, Transplant, Thoracic, and Vascular Surgery, University Hospital of Leipzig, Germany. The study is approved by the Ethical Committee of the Medical Faculty, University of Leipzig (reference number 056/20-ek) and will be conducted in accordance with the principles in the latest revision of the Declaration of Helsinki. Any modifications to the study protocol will require a formal amendment to the protocol. The study has undergone external peer-review by the funding body and is funded by the State Ministry for Higher Education, Research and the Arts, Free State of Saxony, Germany.

### Study group, screening, and recruitment

The patients will be screened, informed, and included in the study at three University Hospitals in Germany (Dresden, Leipzig, and Hannover Medical School). Written informed consent will be obtained from all participants. The target groups of the study are cancer patients with International Classification of Diseases codes C18/19/20 (colorectal cancer), C50 (breast cancer), and C61 (prostate cancer) who underwent curative (R0) surgery at stages T1N0M0 to T3N3M0. Inclusion and exclusion criteria are presented in Tables 1 and 2. The patients will be informed and educated postoperatively before discharge from the hospital about program content and aims, randomization, and the consequences for the control group by the study team or the clinic stuff. Individuals interested in participating will then provide written informed consent after a sufficient period for reflection and further verbal discussion with an investigator. Participant insurance was contracted for study-related harm to study participants.

**Table 1:** Inclusion criteria

#	Inclusion criteria
1	Resected colorectal carcinoma (C18/C19/C20) or breast carcinoma (C50) or prostate carcinoma (C61) up to 6 months after surgery, clinical stages T1–3 and/or N+; M0 according to UICC 8th edition; completed neoadjuvant chemotherapy, and achieved R0 resection
2	Female or Male
3	Age between 18 and 75 years
4	ECOG (Eastern Co-operative Oncology Group) < 1 without acute cardiac, renal, hepatic, endocrine, bone marrow or cerebral disorder
5	Cognitive ability of the patient to understand the postoperative program and to participate actively [26, 27]

**Table 2:** Exclusion criteria

#	Exclusion criteria
1	Complication after tumor resection, which prolong convalescence and may impair adherence to the study interventions
2	Presence of a second malignant tumor (unless curatively treated > 5 years ago)
3	Orthopedic, rheumatological, cardiovascular or neurological (epilepsy, stroke, Parkinson’s disease, muscle wasting diseases such as amyotrophic lateral sclerosis, atrophy, cachexia or multiple sclerosis) contraindications for the exercise program
4	Inability to use the internet or the required applications (tablet, activity tracker, CRPB-TS App)
5	Each active disease, which affects adherence to the study procedures
6	Active alcoholism or illegal drugs consumption within the last six months before study entry
7	Missing patients’ compliance or inability to communicate in German

### Randomization

**Fig. 1:**
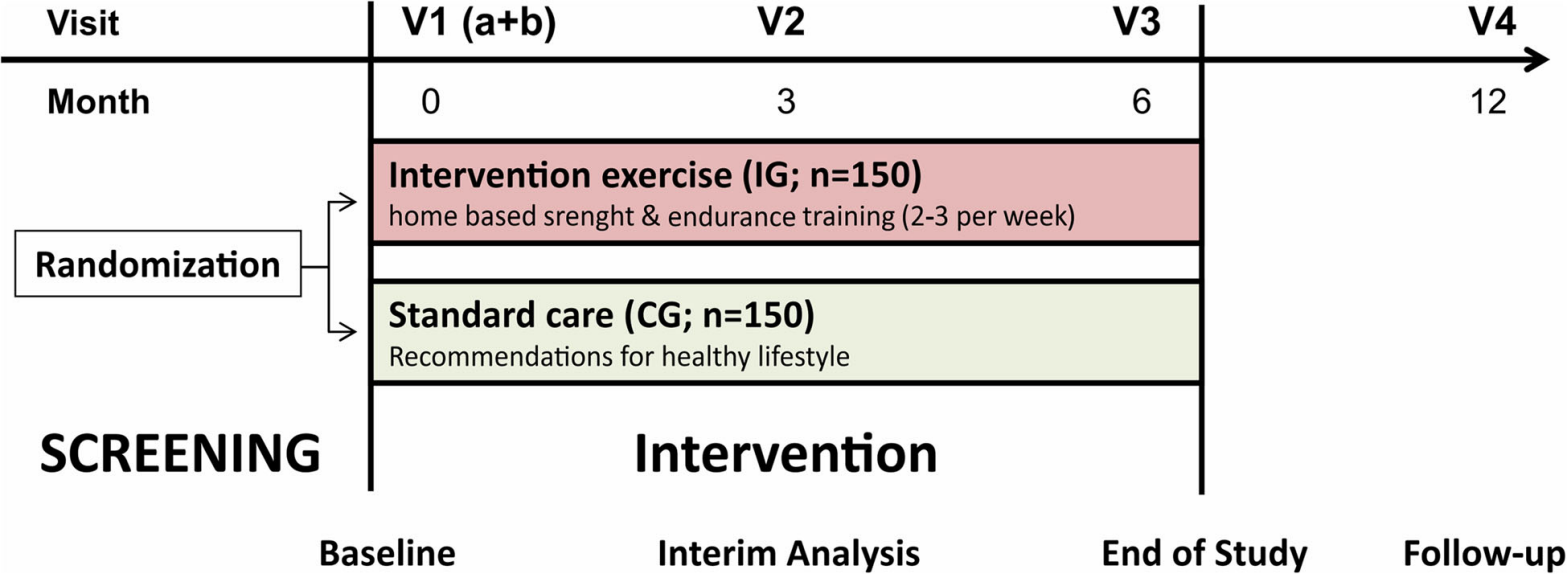
Study flow chart (IG: intervention group, CG: control group, V: visit)

Patients will be randomly assigned to the intervention group (IG) or the control group (CG) following a 1:1 allocation by the Clinical Trial Centre, Leipzig. Randomization will be stratified by using a dynamic minimization algorithm with two factors: the cancer entity (colorectal, breast, or prostate carcinoma) and the gender (only by colorectal carcinoma). Allocation concealment will be ensured by independence of the randomization service and release of the results after recruitment. Recruitment will be spread over 6 months with a subsequent 6-month intervention phase (Fig. 1).

### Objectives and endpoints

The objective of this interventional trial is to determine the effect of a continuously supervised post-surgery online training and lifestyle modification program on patients’ physical and psychological status. Short-term and intermediate-term aims are to increase the cardiopulmonary performance and muscle mass, to reduce abdominal fat, to improve the quality of life, and to reduce depression and fatigue. The recurrence rate and mortality will be addressed in the long term.

The primary endpoint is a 13% increase in the VO_2_ max in the IG when compared with the CG after training for 6 months.

Secondary endpoints after 6 months of online training and lifestyle improvement are as follows.

- Significant decrease in re-hospitalization (decrease by absolute 15%) in the IG when compared with the CG
- Significant improvement in the quality of life, fatigue, and depression (improvement in scores by absolute 10%) in the IG, which is assessed with the European Organization for Research and Treatment of Cancer Quality of Life Questionnaire (EORTC QoLQ-C30, disease specific adaptation)
- Increase in the metabolic rate by 500 kcal (500 Met/min) per week in the IG when compared with the CG
- Increase in patients’ compliance to 95% in the IG
- Improvement/decrease in tumor-associated marker panel

### Sample size calculation

The sample size was calculated according to the expected effect on the primary endpoint (13% increase in VO_2_ max) in the IG when compared with the CG in 6 months [28, 29] for each cancer subgroup. Sample size calculation was based on a power of 0.80 with a two-sided test and an alpha of 0.05. The increase was calculated with respect to the change in the baseline characteristics of IG patients when compared with CG patients, with delta VO_2_ max of 3.6 ml/kg/min, a standard deviation of 5 ml/kg/min, and an average VO_2_ max of 27.5 ml/kg/min at baseline. Thus, the required sample size for each group (IG and CG) of each cancer entity (colorectal, breast, and prostate) was 40. Considering an expected dropout rate of 20%, 50 patients will be recruited (oversampling) for each group and entity. Considering 100 patients (50 in the IG and 50 in the CG) for each cancer entity, 300 patients will be included in the study.

### Study organization

The central components of this new type of care are **c**ross-linked **c**are **p**oints (cCPs), which are located in the respective university hospitals. Their tasks include local project administration (patient recruitment, data collection) and medical health care supply (diagnostics, briefing, telemonitoring support, and cross-sectoral communication). Each cCP will consist of an interdisciplinary team (a physician, a sports scientist, and a cancer nurse). It will care for approximately 100 patients (50 patients each from the CG and the IG).

The medical duties within the cCP will include primary and follow-up care before and after the surgery and in-hospital rehabilitation immediately after the intervention period and 6 month after the intervention. The tasks will include data acquisition, medical supervision of the online program and training, management of patients’ information, structured tumor aftercare in accordance with the respective tumor guidelines, tele-support, and feedback to the referring ambulant physician. The responsibilities of sport scientists will include individual configuration of the intervention program (training and nutrition) and tele-support. The cancer nurses will perform the tasks of patient screening, management of patient information and patient inclusion, monitoring the online program, reporting of vital parameters and clinical chemistry, tele-support, data collection during and after the training, and central data management.

### Cross-sectoral patient file and CRBP-TS application

The organizational and information technology center of the project will include the **e**lectronic cross-**s**ectoral **p**atient file (ESP) and the CRBP-TS application. At baseline, the CRBP-TS mobile application will be downloaded in each participant’s tablet (Lenovo Tab M10 TB-X606X; Lenovo, Hongkong, China) and connected to the activity device (Vivoactive 4; Garmin, Olathe, Kansas, US) via Bluetooth. The activity device will be wear 24 h a day during the entire study period. The CRBP-TS application will be regularly used by the participant to transfer activity tracker and heart rate (chest belt) data via Bluetooth throughout the intervention, to complete questionnaires, and to visualize the home-based training in the intervention group. An automatic data transfer from patient to ESP is provided via an internet access (LTE, Deutsche Telekom AG, Germany) at the tablet divice. The ESP will serve as an electronically case report form (CRF) and will contain the medical report files, patients’ training data, measurements, and manual entries. The data will be prepared and provided to the participating care institutions. Patients will also be able to view their training, fitness, and body composition data.

### Data management and statistical analysis

In the CRBP-TS study, all data will be entered to the ESP at the participating site. All questionnaires will be completed on a dedicated application CRBP-TS and merge to the ESP. There will not be a formal data monitoring committee for this study, however monitoring will be provided by the trail administration stuff, including overall project supervision and progress monitoring. The clinical research assistant will verify all consent forms, compliance with established protocol and procedures, and data quality in the ESP. Following quality control measures, the input data will be locked in the database. Participant files will be maintained in the storage for a period of 10 years after the completion of the study. The access to the ESP will be secured (personal ID and password required) with different levels of security depending on the role of the investigator. The Principal Investigator will be given access to the cleaned data sets.

The evaluation will be performed by an “intent to treat” analysis using all randomized patients in the entity subgroups. For the evaluation, established statistical programs and common statistical analysis methods for longitudinal regression models (such as mixed model with repeated measures) will be used. The intervention arm will be compared with the control arm with respect to all primary analyses and all visits.

### Individual study plan, measurements, and data collection

**Fig. 2:**
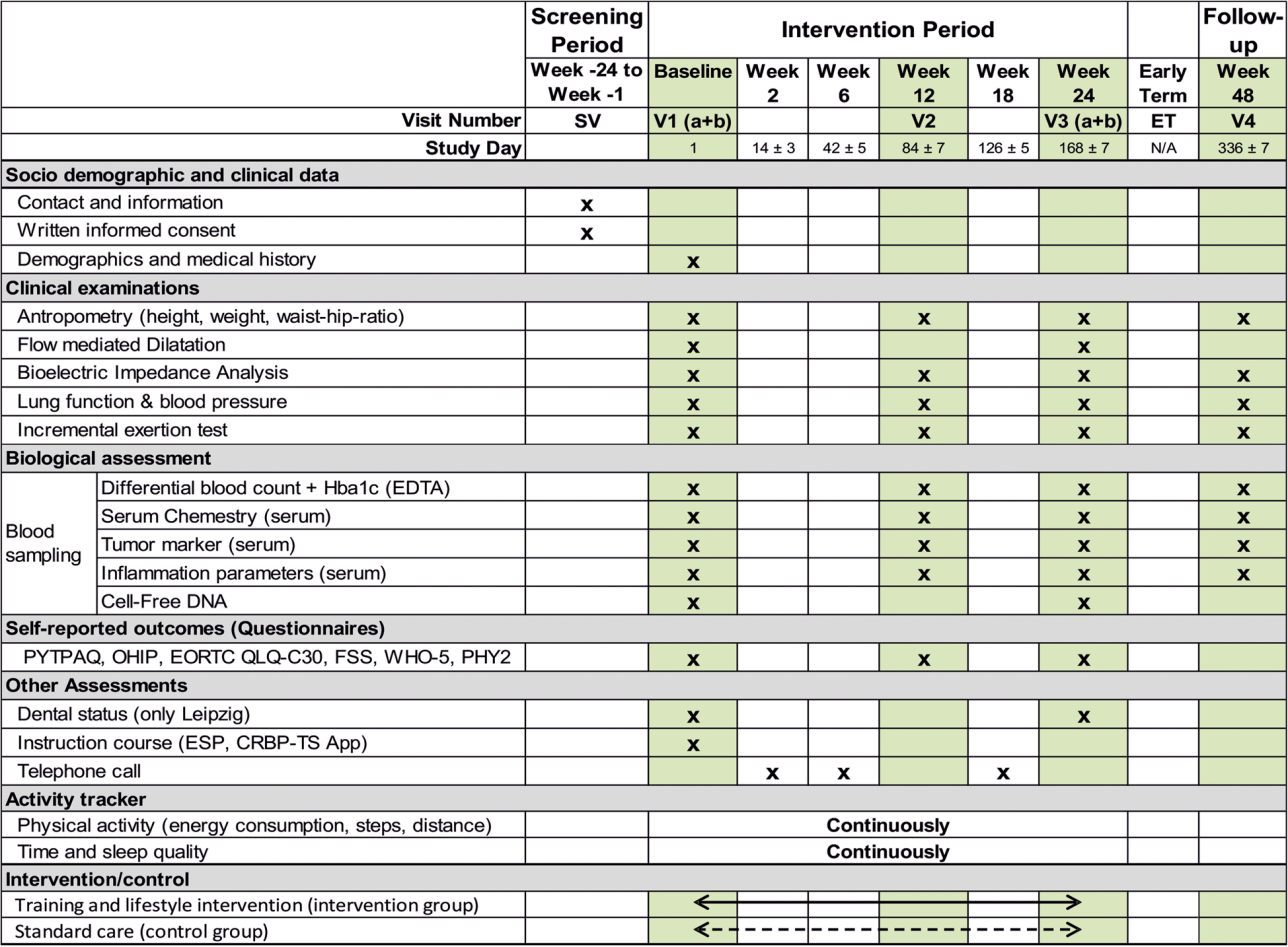
Data collection schedule for the CRBP-TS Study (SV: screening visit; EDTA: Ethylenediaminetetraacetic acid; PYTPAQ: Past Year Total Physical Activity Questionnaire; OHIP: Oral Health Impact Profile questionnaire; QLQ-C30: Quality of Life; FSS: Fatigue Severity Scale; PHY2 & 9: Patient Health Questionnaire; WHO-5: well-being index; ESP: electronical cross-sectoral patient file)

A period of 6 months is planned for the recruitment of participants. The intervention period will be of 6 months. Data collection will be performed at baseline (appointment 1a will include examinations and appointment 1b will include instruction course), at 3 months (appointment 2), and at 6 months (appointment 3, end of the study). After 12 months, a follow-up investigation (appointment 4) will be conducted. The IG will undergo individual training intervention and counseling, while patients from the CG will be provided with standard care as well as comprehensive information regarding health-promoting measures. The daily activity of patients in the IG and the CG will be measured and evaluated by the CRBP-TS application. An overview of the examinations during the study visits is shown in Fig. 2.

The recorded parameters will be stored in the ESP by the cCP or entered by the patients themselves via online questionnaires. To enable the measurement of secondary outcomes, patients from the IG and the CG will receive a wearable device, which will quantify the activity behavior during the entire study period.

At the instruction course (appointment 1b), all patients will receive information about the study program and about the handling of the software and hardware components (tablet with CRBP-TS application, wearable device, instructions about the CRBP-TS application) in small groups.

After 3 months, the first follow-up examination (appointment 2) will be conducted, which will include an incremental exertion test, laboratory analysis (clinical chemistry, tumor markers, inflammation panel), and bioimpedance measurement.

In the final examination (end of the study, appointment 3) after 6 months, all examinations performed at baseline will be repeated.

After 12 months, another follow-up examination (appointment 4) will be conducted in which the measurements from appointment 2 will be repeated.

During the intervention period, physical activity in the IG and the CG will be recorded by a fitness tracker. This assessment will include total calories, resting metabolic rate, number of steps, heart rate (during the training intervention), and sleep quality (length and duration of sleep phases).

In addition, patients will record the intake of their medications using binary confirmation (medications taken or not taken) every morning via the CRBP-TS application. All data will be automatically transferred to the ESP.

The baseline examination (appointment 1a) will include the following measurements.

1. *Incremental exertion test*

Cardiopulmonary exercise testing to assess oxygen uptake (VO_2_) and maximal power output will perform on an electronically braked semi-recumbent ergometer. The initial load will be 30 W with 10 W/min increments until subjective or objective exhaustion or the occurrence of the termination criteria [30]. After the exercise period follows a 5-min recovery period. Measurement parameters will include ventilation, oxygen uptake (breath by breathy analysis, Dynostics, Sicada GmbH, Germany), oxygen saturation, heart rate (electrocardiogram), blood pressure, cardiac output (measured by impedance cardiography, PhysioFlow Q-Link, Manatec Biomedical, France), blood lactate concentration and rating of perceived exertion. Measurement of cardiac output will not be performed in patients with contraindication to impedance measurement (pacemakers).

2. *Endothelial function*

Flow mediated dilation will be measured by the determination of diameter and flow velocity in the brachial artery before and after forearm compression above systolic pressure for 5 min [31].

3. *Anthropometry*

Height and body mass will be used to calculate body mass index and body surface area. Waist circumference will be assessed at the mid-point between the iliac crest and lowest rib according to standard techniques. Segmental bioimpedance measurement will be measured to calculate lean body mass and fat mass.

4. *Clinical chemistry and inflammation marker panel*

Blood sampling will be performed from baseline (V1) to 6 months (V3) to enable translational research and to assess potential predictive factors of outcomes. Ethylenediaminetetraacetic acid plasma, serum and cell-free DNA BCT® will be collected, centrifuged, and stored at − 80 °C until analysis. Inflammatory cytokine levels, immune cell assessment, high-sensitive C-reactive protein, and tumor necrosis factor alpha will be quantified. Adipose and metabolic markers will include hemoglobin A1c, leptin, adiponectin, insulin, and c-peptide.

5. *Tumor markers and liquid biopsies*

Tumor entity-specific microRNA, circulating free DNA, and circulating tumor DNA; prostate-specific antigen; carcinoembryonic antigen; carbohydrate antigen 15–3; and carbohydrate antigen 19–9 will be determined.

6. *Questionnaires*

The questionnaires will include entity-specific quality of life (EORTC QoLQ-C30), depression (yes/no type Patient Health Questionnaire-2 and Depression Anxiety Stress Scale), and fatigue (Fatigue Severity Scale) risk screening for colorectal, breast, and prostate cancer. Preoperative leisure activity (Past Year Total Physical Activity Questionnaire), WHO-5 (well-being index) and dental care/periodontal status (Oral Health Impact Profile questionnaire) will also be recorded.

### Intervention

#### Intervention group

Using the CRBP-TS application, patients from the IG will receive a complex training and lifestyle improvement program. The exercise training will consist of indication-specific, individualized, heart rate-limited, and online-supported strength-endurance home training with primary focus on improving oxygen capacity using video presentations calibrated according to oxygen consumption. Individual entry and follow-up levels will be used. The follow-up levels will be based on patients’ individual performance development measured by their wearable devices. All exercise adaptions will be supervised by the cCP team.

Details of the training intervention (2–3 times per week for 30 min) are as follows.

- Individualized video-home training with feedback information to patients and the cCP: The strength-endurance training based on the initial examination using an online video presentation with indication-specific focuses. Four rounds with five different body-weight exercises (upper and lower body) with 40 s loading and 20 s of recovery phase will be performed. Between rounds there will be a 60 s break. The intensity of the exercise is individually adjustable. Training videos will be changed and modified weekly to provide training variety, and progression.
- Individualized outdoor endurance training: Depending on patients’ interests supervised endurance training (such as jogging, walking, cycling, fitness facilities, rehabilitation sports) with activity recording and heart rate monitoring should be implemented.

Details of the lifestyle intervention and patient empowerment are as follows.

- Structured information on general health improvement, disease prevention, and lifestyle changes (diet, exercise, anti-inflammatory prophylaxis, new therapy options, fatigue, depression, and self-perception) provided by the cCP team via the CRBP-TS application.
- Advice and appointment reminders (such as reminders about taking medication or failed training) via short message service or e-mail
- Online feedback of key parameters (training success in strength and endurance training, performance, heart rate)

During the 6-month intervention, patients will have regular appointments with their outpatient physicians. All physicians will be able to access the ESP and the data summaries including the online monitoring and training data. The outcomes will be discussed with the patients if needed. An interdisciplinary online conference will be conducted every 4 months, which will include family doctors, medical specialists, and the cCP team.

#### Control group

Patients in the CG will receive standard care over a period of 6 months. After inclusion, they will receive general information about necessary lifestyle changes according to the current guidelines. Further course of the study will be analogous to the course followed by the IG.

### Safety analyses

The safety assessment includes observation and recording of any grade of adverse events (AE) and serious adverse events (SAE) during the study, as well as the results of laboratory analyses and clinical examination. Participants in the exercise training group will also be asked about the occurrence of an AE at each training session. The researchers are responsible to take appropriate measures for the AE and determine the causal relationships between the adverse events and the exercise intervention.

## Discussion

The main objective of this prospective randomized controlled study is to evaluate the systematic use of a home-based physical training and lifestyle intervention module with a multidirectional online monitoring tool supervised by specific clinic staff. Monitoring will include therapy adherence, physical and mental capacity, and efficiency of lifestyle changes. The exemplary care will be planned and continuously adapted at the cCPs consisting of specialized staff (doctor, cancer nurse, sports scientist) based on the ESP files that include findings, training, and measurement data.

Several studies on colorectal, breast, and prostate cancer patients have shown that physical training results in a 20–40% relative risk reduction for recurrence and tumor-associated mortality [6–12]. Therefore, guidelines consistently include the systematic use of physical training in postoperative therapy, with high relevance for the majority of the tumor entities. A balanced combination of strength and endurance training would be highly effective in reducing short-term and intermediate-term tumor-associated morbidity, recurrence, and metastases [18]. Positive effects have also been demonstrated for general cancer symptoms and comorbidities [13, 15, 17]. The existing literature primarily consists of questionnaire surveys based on instructions for lifestyle changes and qualitative implementation of the training programs. Despite the protective, therapeutic, and regenerative efficiency of physical training, a systematic implementation strategy is still lacking. Although physical training also results in significant improvement in several comorbidities, this effective therapy option is currently not used to its full potential.

Essential factors for motivation and high adherence to a training intervention include constant supervision, individual dosage based on personal requirements (overweight and joint problems), and fewer impediments (distance, shame). The best results can be expected from a permanent connection between instructors and patients, preferably online. A supervised program guidance is beneficial for most patients [32]. Additionally, patients should also be able to follow their performance development and clinical findings to maintain motivation. This requires bidirectional online communication, a comprehensible presentation of all findings, and telephone communication with patients if necessary. Objective activity monitoring must be integrated into the process.

CRBP-TS address these issues and offer a cross-sectoral implementation of monitoring, training, and lifestyle (patient empowerment) based on a comprehensive online application (CRBP-TS application). This care structure is independent of patients’ individual living conditions and offers safety and efficiency even in rural areas. In addition to the home-based online intervention, the CRBP-TS study includes comprehensive general and tumor-specific diagnostics (cardiopulmonary performance, liquid biopsy, metabolism, tumor marker panel, circulating tumor DNA, circulating microRNA, and cytokines).

Current studies have shown a correlation of periodontitis with an increased general [33–35] and entity-specific mortality risk for breast, colon, rectum, and prostate carcinomas [34, 36–42]. Therefore, dental screening is also a part of the present study.

To the best of our knowledge, this is the first randomized and prospective study focusing on cardiovascular and metabolic outcomes as well as molecular genetic effects based on the quantified dosage of therapeutic training for CRBP tumor entities. Due to increasing incidence of malignant diseases, poor prognosis, and considerable impairment in the quality of life, there is a need for improvement in cancer care. CRBP-TS project is of economic as well as of social and ethical importance. Due to the short observation period of 6 months and the correlation between mortality and exercise capacity, VO_2_ max will be used as the primary endpoint. Mortality and recurrence rate will be observed as secondary endpoints, requiring longer follow-up.

### Trial status

Protocol version - Date and version identifier: Issue Date 30 March 2020.

The recruitment started in August 2020 and will be approximately completed in June 2021.

## Data Availability

The datasets generated during the present study can be obtained from the corresponding author on reasonable request. The trail results will be communicated via publications.

## Abbreviations

AE: Adverse event
cCP: Cross-linked care points
CG: Control group
CRBP-TS: Colorectal, Breast, and Prostate Cancer - Telemonitoring and Self-management
CRF: Case report form
DNA: Deoxyribonucleic acid
EDTA: Ethylenediaminetetraacetic acid
EORTC: European Organization for Research and Treatment of Cancer
ESP: Electronic cross-sectoral patient-file
FSS: Fatigue Severity Scale
IG: Intervention group
kcal: Kilocalorie
LTE: Long Term Evolution
min: Minute
OHIP: Oral Health Impact Profile
PHY2 & 9: Patient Health Questionnaires
PYTPAQ: Past Year Total Physical Activity Questionnaire
QoLQ-C30: Quality of life questionnaire
RNA: Ribonucleic acid
SAE: Serious adverse event
VO_2_max: Maximal oxygen uptake
WHO-5: World Health Organization well-being index
